# Technology and Innovation in Global Ophthalmology: The Past, the Potential, and a Path Forward

**DOI:** 10.1097/IIO.0000000000000450

**Published:** 2022-12-27

**Authors:** Travis K. Redd, Tala Al-Khaled, R.V. Paul Chan, J. Peter Campbell

## Introduction

The World Health Organization estimates that at least 2.2 billion people suffer from visual impairment globally.^1^ Nearly half of these cases are preventable or undertreated. Limitations in the accessibility and quality of vision care remain significant drivers of this burden of disease. These issues are important in every community regardless of socioeconomic status but disproportionately affect individuals in low-income and middle-income countries. “Accessibility” in this context refers to the ability of a person in need to contact, either virtually or in person, an appropriate care provider capable of diagnosing and managing the underlying etiology of their visual impairment. A number of barriers can limit accessibility, including the cost of receiving care, the physical distance between patient and provider and lack of feasible transportation to traverse this distance, communication limitations, including language and cultural barriers between provider and patient, lack of individual agency or a sense of not being a participant in one’s care, and an inadequate number of qualified providers relative to the burden of illness in the population. “Quality” of care refers to the amount of benefits patients receive once they make contact with an appropriate eye care provider. Primary barriers to high-quality care include a lack of infrastructural support, limited provider education, and inadequate availability of effective diagnostic and therapeutic interventions.

Herein we provide a broad overview of historic successes in the quest to improve the accessibility and quality of eye care globally (“the past”), opportunities for integrating recent technological advances such as AI (“the potential”), and “a path forward” to successfully develop and implement these innovations in a responsible and impactful manner.

## The Past

The global eye care community has a rich history of innovating care delivery models to address unmet needs in vision health. For example, despite effective treatment, cataract remains the leading cause of preventable blindness worldwide. Efforts to reduce the cost of performing cataract surgery resulted in the development of a novel surgical approach, manual small incision cataract surgery, which achieves comparable outcomes to state-of-the-art phacoemulsification cataract surgery at a fraction of the cost.^2^,^3^ Novel care delivery systems have enabled large-scale implementation of this surgical technique in areas of high need. For example, the Aravind Eye Care System in India and Tilganga Eye Centre in Nepal have been remarkably successful in delivering high-volume, high-quality cataract surgery to regions with very high disease burdens.^4^,^5^ The combination of cost-saving surgical techniques and community outreach allows institutions such as these to achieve sufficient surgical volume to benefit from economies of scale. Care delivery models founded on these principles have been replicated in geographically diverse environments with similar success.^6^

Another major barrier to accessible care is the physical distance between patients and care providers possessing the skills, knowledge, capacity, and infrastructure to provide needed eye care. Several approaches have been employed to close this distance gap. Surgical outreach camps can bring providers and supplies to populations in need, while educating and supplying local providers may provide a more sustainable alternative to reduce regional accessibility gaps. For example, organizations like ORBIS and Seva have successfully trained and equipped 10s of 1000s of eye care professionals globally.^7^

Teleophthalmology bridges the distance gap by virtually connecting patients and providers and has been shown to improve accessibility to eye care, particularly in low-income and middle-income countries.^8^ This concept can be implemented in a number of ways, for example, by providing live consultation with a remote expert for real-time diagnostic and therapeutic guidance, delayed remote expert review of imaging for large-scale screening interventions, or using virtual language interpreter services to ameliorate language and (to some extent) cultural barriers between provider and patient. Moreover, teleophthalmology, by extension, includes the concept of tele-education. This refers to the development of web-based platforms that provide standardized training tools to build providers’ knowledge base and enhance diagnostic competency and clinical management. Most current programs consist of self-paced training, preparing local clinicians and technologists with the ability to (at minimum) triage treatment-requiring cases without the need to rely on remote providers.^9^

The successes of the global ophthalmology community to date emphasize that overcoming the barriers to accessible, high-quality eye care for all can be achieved using a framework that adapts existing approaches (or develops de novo health care models) with an emphasis on scalability, sustainability, cultural competency, and stimulating patients’ individual agency and sense of empowerment.

## The Potential

While many of the above interventions have been remarkably impactful, recent technological advances have resulted in significant enthusiasm for potential novel approaches to address global vision issues.^10^ Computer processing power continues to grow at an exponential rate (ie, Moore’s law has proven prescient), and cloud computing, coupled with the ubiquity of internet-connected devices, has enabled information processing capabilities not previously achievable.^11^ Simultaneously, the quantity of health data continues to increase, both in structured formats (eg, electronic health records, clinical data registries such as the IRIS registry, and claims databases) and unstructured formats (eg, medical imaging, electrocardiograms, electroencephalograms, wearable health devices, etc.). The past decade has also seen methodologic revolutions in artificial intelligence (particularly the advent of deep learning), big data analytics, and informatics, suggesting the opportunity to adapt these new methodologies to the ever-expanding corpus of health data to improve eye care accessibility, quality, and our scientific understanding of eye diseases.

### Improving Global Eye Care Accessibility and Quality

Nowhere is the potential impact of advancing technology on global vision health more apparent than in the concept of large-scale automated image-based screening for eye diseases. The burden of many major global causes of blindness far exceeds the capacity of traditional telemedicine approaches with remote expert interpretation for secondary prevention. The application of artificial intelligence extends the concept of telemedicine beyond connecting patients to care providers, allowing the potential for massive scalability through assistive diagnosis or autonomous screening. There are now 2 FDA-approved devices for automated detection of diabetic retinopathy, and similar success may be achievable for other major global causes of preventable or treatable blindness, including glaucoma, macular degeneration, and retinopathy of prematurity.^12^–^15^ Narrowing the population of patients requiring in-person evaluation and management with an expert care provider would allow much more effective utilization of limited health care resources.

Artificial intelligence (AI) and clinical informatics may also provide benefits for tertiary prevention purposes. For example, clinical decision support systems have long been leveraged to reduce prescribing errors.^16^ Similar point-of-care systems capable of machine learning may leverage the quantity of data contained in electronic health records to enable more accurate risk stratification of patients with eye diseases, quantitatively monitor therapeutic response, and/or provide supplemental information to guide treatment decisions. In addition, advances in bioinformatics have enabled the massive amount of information encoded in genetic samples to be processed and meaningfully interpreted, which may provide novel risk modeling and diagnostic capabilities. As an example of the latter, deep sequencing methods have shown potential for accurate, hypothesis-free identification of ocular infections, with a much broader diagnostic range than traditional primer-based techniques such as PCR.^17^

Finally, artificial intelligence may also be utilized to address language barriers between the provider and the patient. Automated interpretation of speech and language has demonstrated remarkable progress in recent years, with some models capable of accurate interpretation between 100s of languages (eg, Google Translate). This may provide a similar scalability benefit compared with traditional in-person or remote human interpretation between the provider and patient, although the accurate interpretation of medical speech remains a challenge for modern AI models.^18^

### Improving Scientific Understanding of Eye Disease

Technology, in addition, provides opportunities to gain insight into disease characteristics, including epidemiology, genetic factors, clinical presentation, and response to treatment, which can inform the revision of what is considered “state-of-the-art” eye care. Structured data in clinical registries such as the IRIS registry, analyzed using tools and best practices within the framework of clinical informatics and big data analytics, provide opportunities to gain knowledge of diseases that would not be feasible with traditional epidemiologic methods.^19^ Similarly, leveraging the massive amount of unstructured data available on the internet can provide an opportunity to conduct large-scale epidemiologic surveillance of various diseases (eg, determining temporal and geographic patterns in the epidemiology of infectious conjunctivitis by analyzing Google search trends).^20^

## A Path Forward

While the pace of innovation in this field is exciting, several significant barriers remain before tangible benefits can be obtained from many of these applications of artificial intelligence and information technology to global eye health. Most modern deep learning models require large amounts of accurately labeled training data to achieve high performance, but “FAIR” (findable, accessible, interoperable, and reusable), machine-learning-ready medical data are scarce.^21^ For example, computer vision (the AI sub-field dedicated to image interpretation) is the primary area of enthusiasm for applications of AI to ophthalmology due to the importance of image-based diagnosis in our field. However, ocular images are often stored on local devices in proprietary file formats, with variable adherence to Digital Imaging and Communications in Medicine standards. This makes the collation of datasets from multiple devices and/or institutions highly problematic. As the American Academy of Ophthalmology recently suggested, stricter standardization of ocular image file formats and adherence to Digital Imaging and Communications in Medicine standards could significantly reduce the scarcity of FAIR imaging data for machine learning applications.^22^ The “Bridge2AI” program recently implemented by the National Institutes of Health similarly emphasizes the importance of generating large-scale, privacy-preserving FAIR data to advance the applications of AI and big data in medicine.^23^

Even after sufficient data has been accumulated and an AI model has been successfully trained and evaluated, there remain significant barriers to implementing such models in the health care setting and achieving benefits to patients. There is a striking discordance between a large number of AI publications in ophthalmology journals and the relatively low number of systems in deployment.^24^ This phenomenon is not isolated to ophthalmology but rather is known generally as the “implementation gap” of machine learning in health care.^25^ There are several reasons for this gap, including a lack of reimbursement models for these devices, ethical concerns, including demonstrable bias in AI algorithms currently in deployment, and patient and provider hesitancy towards the adoption of AI models in clinical care. Overcoming these obstacles ought to be our goal and will require a partnership between multiple stakeholders, including computer and data scientists, clinicians, investors, health systems, and the public.

## Conclusions

The potential exists for recent technological advances to transform the way ophthalmic care is delivered around the world and reduce preventable blindness. To bring these technologies to patients, we need to: (1) Learn from prior successes in global ophthalmology by focusing on community engagement, building sustainable infrastructure, and optimizing care delivery models; (2) Fund research to support the development of FAIR datasets that accurately represent diverse populations and reduce the machine learning implementation gap; and (3) Foster active collaboration among all stakeholders, including patients, clinicians, public health experts, machine learning engineers, informaticists, data scientists, and implementation scientists.

The low-hanging fruit in blindness prevention has always been the implementation of existing knowledge. Although there are many high-tech innovations in ophthalmic imaging, constantly evolving surgical techniques, and new drugs, the most impactful interventions in the next few decades may be in the realm of implementation science, solving barriers such as data infrastructure, adapting care models to local contexts, and creating scalable and sustainable revenue models for technological innovations.^26^ The global vision community has a long history of overcoming barriers to in providing high-quality eye care to the medically underserved. We are optimistic the enthusiasm of this community will achieve similar success in navigating these novel technological innovations and look forward to increasing the accessibility and quality of eye care available to patients in need.
